# Shuffling Improves the Acute and Carryover Effect of Subthalamic Coordinated Reset Deep Brain Stimulation

**DOI:** 10.3389/fneur.2022.716046

**Published:** 2022-02-18

**Authors:** Jing Wang, Sinta P. Fergus, Luke A. Johnson, Shane D. Nebeck, Jianyu Zhang, Shivaputra Kulkarni, Hemant Bokil, Gregory F. Molnar, Jerrold L. Vitek

**Affiliations:** ^1^Department of Neurology, University of Minnesota, Minneapolis, MN, United States; ^2^Boston Scientific Neuromodulation, Valencia, CA, United States

**Keywords:** Parkinson's disease, deep brain stimulation, subthalamic nucleus, coordinated reset, non-human primates

## Abstract

Coordinated reset deep brain stimulation (CR DBS) in the subthalamic nucleus (STN) has been demonstrated effective for the treatment of the motor signs associated with Parkinson's disease (PD). A critical CR parameter is an order in which stimulation is delivered across contacts. The relative effect of alternating vs. not alternating this order, i.e., shuffling vs. non-shuffling, however, has not been evaluated *in vivo*. The objective of this study is to compare the effect of shuffled vs. non-shuffled STN CR DBS on Parkinsonian motor signs. Two Parkinsonian non-human primates were implanted with a DBS lead in the STN. The effects of STN CR DBS with and without shuffling were compared with the traditional isochronal DBS (tDBS) using a within-subject design. For each stimulation setting, DBS was delivered for 2 or 4 h/day for 5 consecutive days. The severity of PD was assessed using a modified clinical rating scale immediately before, during, and 1 h after DBS, as well as on days following the discontinuation of the 5 days of daily stimulation, i.e., carryover effect. Shuffled STN CR DBS produced greater acute and carryover improvements on Parkinsonian motor signs compared with non-shuffled CR. Moreover, this difference was more pronounced when more effective stimulation intensity and burst frequency settings were used. tDBS showed limited carryover effects. Given the significant effect of shuffling on the effectiveness of CR DBS, it will be critical for future studies to further define the relative role of different CR parameters for the clinical implementation of this novel stimulation paradigm.

## Introduction

High frequency isochronal “traditional” deep brain stimulation (tDBS) is an established treatment for the motor signs associated with Parkinson's disease (PD). Its effectiveness, however, can be limited due to side effects resulting from unwanted current spread into adjacent fiber pathways, such as the internal capsule or non-motor regions of the targeted structure (1–3). The concept of coordinated reset (CR) stimulation arose from computational modeling studies that suggested that low amplitude periodic stimulation of synchronized neuronal populations can induce a long-lasting desynchronizing effect (4, 5). CR DBS alternates stimulation across multiple contacts of the DBS lead and is hypothesized to induce a desynchronizing effect at lower current intensities than that required with tDBS (6, 7). CR DBS has been shown in both preclinical and clinical studies to produce acute motor improvement similar to tDBS with the added benefit that motor improvement would persist for hours, days, or weeks following discontinuation of stimulation, i.e., carryover effect (8–10). It may reduce the incidence of side effects by minimizing the current spread.

**Figure 1:**
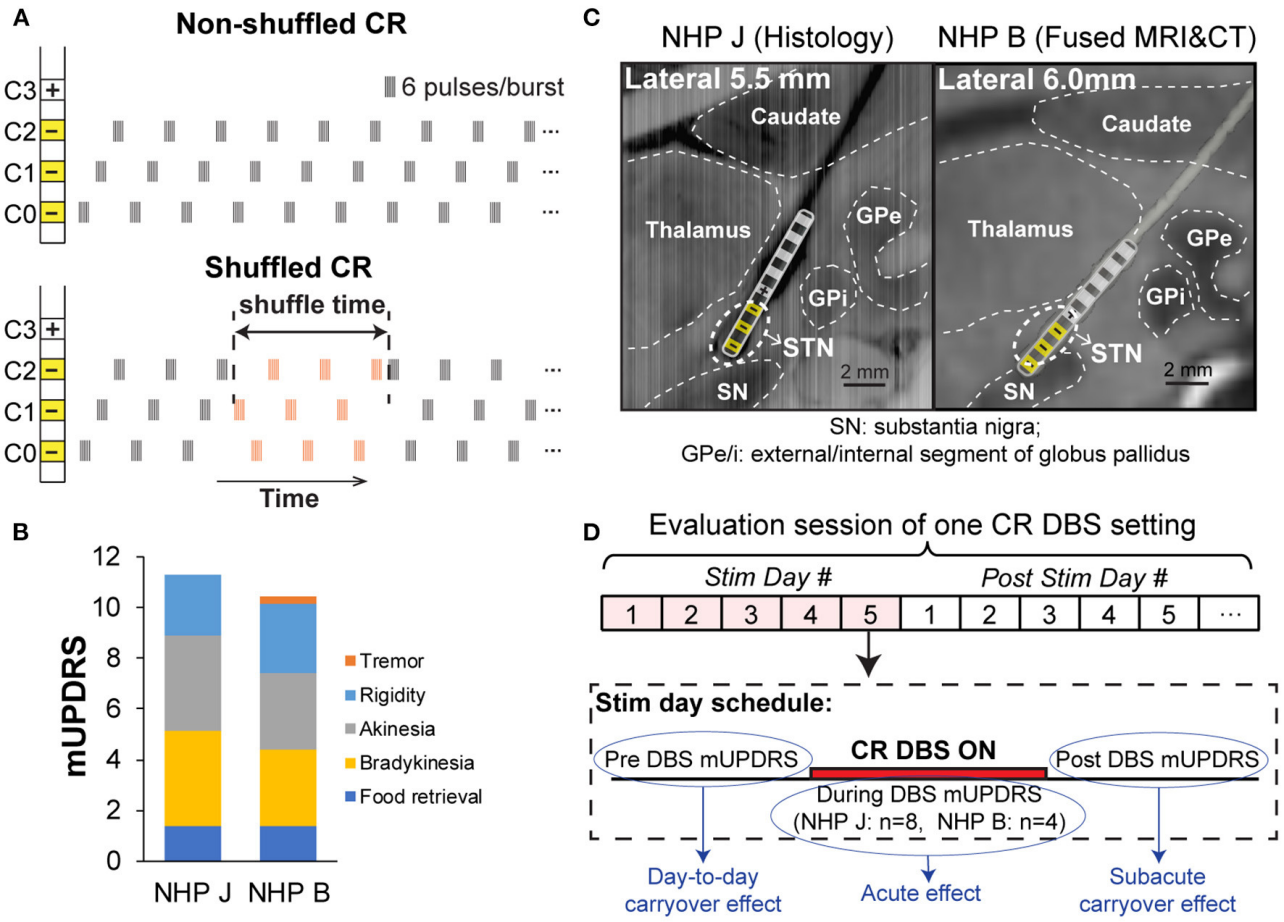
**(A)** Non-shuffled and shuffled coordinated reset deep brain stimulation (CR DBS) patterns. Top: Non-shuffled CR DBS repeats the order of delivering bursts to different contacts, i.e., same contact sequence, throughout the stimulation time. Bottom: Shuffled CR pseudo-randomly changes the contact sequence at a certain frequency. **(B)** Mean total and subscores of modified Unified Parkinson's Disease Rating Scale (mUPDRS) for two Parkinsonian monkeys. **(C)** Deep brain stimulation (DBS) lead locations. Left: Sagittal view of the DBS lead track reconstructed from histology in NHP J (C0-C7 from bottom to top contact); Right: Fused MRI/CT images showing the DBS lead location. The lead schematic was overlaid in each figure and the stimulation contacts for CR DBS are indicated (yellow). **(D)** The experiment timeline demonstrates the evaluation schedule of CR DBS, and the mUPDRS scores used to assess the acute, subacute carryover, and day-to-day carryover effects.

Coordinated reset stimulation was originally designed to deliver stimulation at multiple locations within the target structure by stimulating through individual contacts of a DBS lead using a repeated sequence of contacts (Figure 1A, top), i.e., non-shuffled pattern (4, 11–13). It was hypothesized that the effectiveness of this non-shuffled pattern results from activating neuronal subpopulations in a phase-shifted manner leading to a desynchronizing effect (14). In subsequent computational studies, however, a pseudorandomized sequence of contacts was used (Figure 1A, bottom), i.e., shuffled pattern, and this sequence was found to more effectively desynchronize neuronal populations than the non-shuffled CR (5). Although shuffled CR patterns have been used in recent studies (8–10, 15–17), the relative effect of these two approaches has not been investigated *in vivo*. In this study, we present a case series of two Parkinsonian non-human primates (NHPs) where we explored the relative effect of shuffled vs. non-shuffled subthalamic nucleus (STN) CR DBS using a within-subject design. We hypothesized that shuffled STN CR DBS would produce a greater acute effect as well as a longer carryover effect on motor improvement when compared with the non-shuffled CR DBS. Our findings provide preliminary evidence supporting this hypothesis and support the concept that shuffling the pattern of contact stimulation is an important feature of CR stimulation.

## Materials and Methods

Animal care complied with the Guide for the Care and Use of Laboratory Animals and all procedures were approved by the Institutional Animal Care and Use Committee.

### Animals and Motor Assessment

The 1-methyl-4-phenyl-1,2,3,6-tetrahydropyridine (MPTP) NHP model of PD was used in this study. Two adult female rhesus monkeys (NHP J, 10 kg; NHP B, 8 kg) were implanted with a DBS lead in the STN using an approach similar to that was described in previous studies (9, 18). Briefly, a cephalic chamber was placed on the skull oriented to target the STN in each animal. Microelectrode recording and stimulation techniques (19) were used to map the sensorimotor region and borders of the STN, following which an 8-contact DBS lead (NuMed Inc., TX, USA, 0.63 mm diameter, 0.5 mm contact height, and 0.5 mm space between contacts) was implanted. A version of the Unified Parkinson's Disease Rating Scale modified for NHPs (mUPDRS) (9, 20, 21) was used to assess the severity of parkinsonism on the side contralateral to the site of DBS implantation in each animal and changes in motor signs under different conditions of stimulation. The mUPDRS consists of scores for rigidity, akinesia, bradykinesia, and tremor for the upper and lower limbs, and food retrieval on a 0–3 scale (0 = unimpaired, 3 = severe); maximum total score = 27. The animals were rendered moderately Parkinsonian (mUPDRS: 10–17) using the neurotoxin MPTP. The mUPDRS scores for each animal were 11.3 ± 0.7 (mean ± SD, *n* = 22) in NHP J and 10.4 ± 0.4 (*n* = 22) in NHP B (Figure 1B). Both NHPs demonstrated akinesia, rigidity, and bradykinesia, while tremor was minimal and intermittently observed in both animals. Following completion of the study, NHP J was euthanized, and histology was performed. The 40 μm coronal sections were imaged and visualized in Avizo (FEI) with the sagittal view extracted to show the DBS lead location (Figure 1C, left). For NHP B, a post-implant CT was merged with the pre-operative MRI to verify the location of lead (Figure 1C, right). The schematic of the lead was created from an image of the lead that was overlapped with either the histologic lesion induced by the lead (Figure 1C, left) or the CT reconstruction (Figure 1C, right). This was used to demonstrate the location of the lead and individual contacts within the subcortical target.

### Experiment Protocol

Prior to DBS testing in each animal, the mUPDRS assessment was performed 10 times over 2 weeks in NHP J and 14 times across 4 weeks in NHP B, to establish the baseline severity of motor signs for the study. Within each evaluation session (Figure 1D), CR DBS was delivered with an implantable pulse generator (IPG) (Precision Spectra, Boston Scientific, MA, USA, constant current) for 4 (NHP J) or 2 (NHP B) h daily for 5 consecutive days. This intermittent delivery pattern (2–4 h of stimulation per day) was determined based on previous modeling (22) and *in vivo* studies (8–10). The daily stimulation duration was set as 4 h originally for both NHPs based on our previous study (9), but reduced to 2 h for NHP B as the mUPDRS plateaued within 2 h of stimulation in this animal. mUPDRS scores were obtained daily on stimulation days pre, every 30 min during, and at 60 min post-DBS, and once every afternoon for at least 5 days following the end of 5 stimulation days. A new evaluation session (Figure 1D) was not initiated until the mUPDRS score returned to baseline. tDBS was delivered with a different IPG (Soletra, Medtronic, Ireland, constant voltage) for NHP J as the Boston Scientific IPG used for CR DBS was not available at the time, the experiments were initially performed. For NHP B, tDBS was delivered with the Boston Scientific IPG. tDBS was evaluated using the same schedule as with CR DBS, with the exception that the mUPDRS scores obtained during stimulation were collected every hour (1st, 2nd, and 3rd h in NHP J; 1st and 2nd h in NHP B) rather than every 30 min. Following 5 days of tDBS, mUPDRS scores were obtained for only 2 days for NHP J and were not further assessed for NHP B as the mUPDRS returned to baseline within minutes following discontinuation of tDBS in both animals.

Therapeutic stimulation parameters for tDBS were determined using a standard monopolar review (1). tDBS was delivered through C0–/C2+ at 2.1 V in NHP J, and C1–/C2+ at 0.32 mA in NHP B, with a pulse width of 120 μs and frequency of 130 Hz. For CR DBS, the four contacts within the STN region (C0/C1/C2–, C3+) were selected based on the lead location combined with results from the monopolar review in both NHPs. CR stimulation parameters were selected based on previous studies (8, 9), referred to as the “default” setting, including 0.1 mA intensity, 120 μs pulse width, 6 pulses/burst, 150 Hz intra-burst rate, and 21 Hz burst frequency. Shuffling (Figure 1A) was performed manually every 30 min in NHP J as the device was not capable of shuffling automatically at the time of the experiment. For NHP B, the function of automatic shuffling was incorporated into the Boston Scientific IPG and shuffling was performed automatically with a shuffling interval of 10 s. In NHP B, additional shuffled CR DBS sessions were performed to explore the impact of different current intensities and burst frequencies on the therapeutic effect. A stimulation intensity (0.16 mA) and burst frequency (27 Hz) that induced greater acute motor benefits and longer carryover effect relative to the default setting (0.1 mA, 21 Hz) was identified in this animal. Both shuffled and non-shuffled sessions were performed using these settings to further compare the effect of each on motor signs.

### Data Analysis

All the mUPDRS scores obtained during DBS (acute scores) were converted into the percentage improvement relative to the baseline score: percentage improvement in mUPDRS = 100^*^(baseline score – acute score)/baseline score. The baseline score was defined as the median of the baseline mUPDRS scores obtained prior to DBS testing (10 scores for NHP J and 14 scores for NHP B). The daily acute effect of CR DBS was determined by the median percentage improvement in the mUPDRS scores obtained during daily CR DBS (Figure 1D, *n* = 8 for NHP J and *n* = 4 for NHP B each day). The acute effect of tDBS was determined by the median percentage improvement in the mUPDRS scores obtained during tDBS across the 5 stimulation days (*n* = 15 for NHP J and *n* = 10 for NHP B), due to the limited number of scores obtained each day and a similar level of effect across days. The acute effect of tDBS and daily acute effect of CR DBS were compared with the baseline using the Steels test with control = baseline following a Wilcoxon test [χ^2^ (DoF,N)]. Similarly, the daily acute effect of CR DBS was compared with the acute effect of tDBS using the Steels test with control = tDBS. The daily acute effects of shuffled and non-shuffled CR DBS were also compared with each other using the Wilcoxon test. Statistical analyses were performed in JMP (SAS Institute Inc., NC, USA), and alpha was corrected for 26 comparisons using the Bonferroni method. Detailed results of the statistical analysis are shown in Supplementary Table 1. The sub-acute carryover effect was determined by the percentage improvement in the mUPDRS score obtained 60 min post-DBS (1 score per stimulation day), relative to the pre DBS score on stimulation day 1. The day-to-day carryover effect was determined by calculating the percentage improvement in the pre DBS mUPDRS scores on stimulation days 2–5 and each day following discontinuation of the 5 days of CR DBS (1 score per day), relative to the pre DBS score on stimulation day 1.

## Results

### Non-Human Primate J

**Figure 2:**
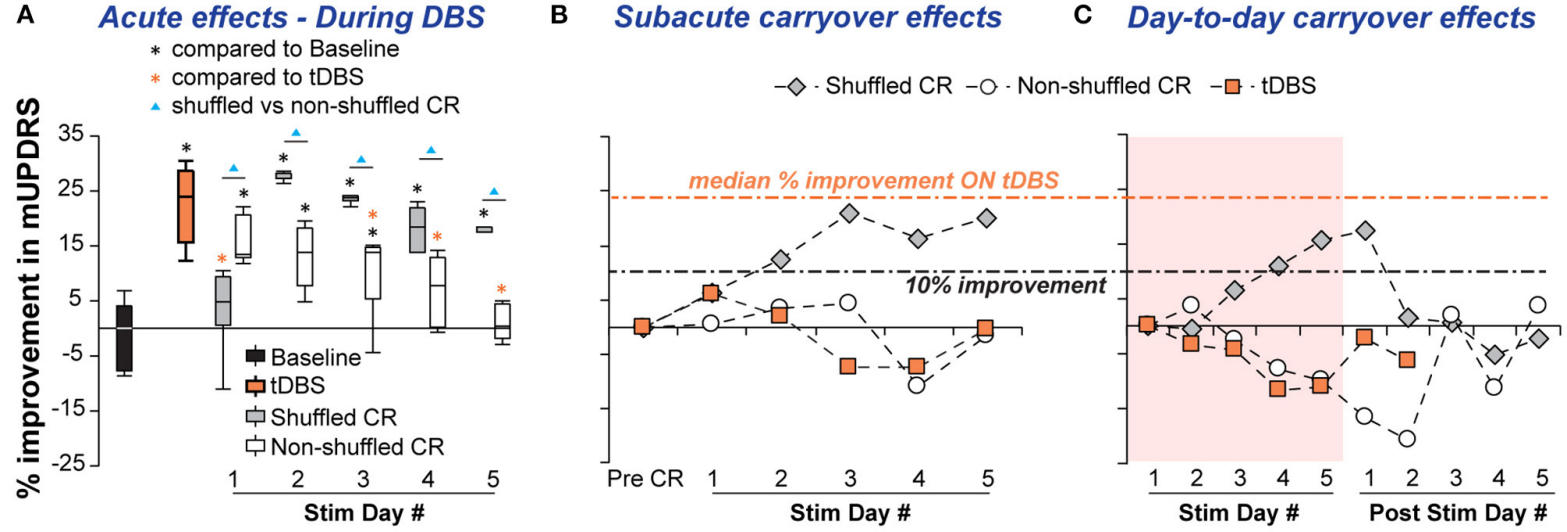
Acute and carryover improvements in mUPDRS acquired by shuffled and non-shuffled CR DBS in NHP J. **(A)** Acute improvements were compared with the baseline using the Steel's test with control = Baseline (*p < 0.05) following a Wilcoxon test. Daily acute improvements of CR DBS were also compared to traditional DBS (tDBS) using the Steel's test with control = tDBS (*p < 0.05). Within each stimulation day, the shuffled and non-shuffled CR DBS were compared using the Wilcoxon test (▴*p* < 0.05). **(B)** Subacute carryover effects of CR DBS indicated by the percentage improvement in the post-DBS mUPDRS. **(C)** Day-to-day carryover effects of CR DBS indicated by the percentage improvement in the daily morning mUPDRS on stimulation and post-stimulation days.

Daily acute effects: except for a few days, both shuffled and non-shuffled CR DBS produced significant acute motor improvement. The degree of improvement during shuffled CR was greater than non-shuffled CR except for stimulation day 1 (Figure 2A). Shuffled CR DBS produced acute improvement comparable with that produced by tDBS except for stimulation day 1, while the non-shuffled CR produced significantly less acute benefit than tDBS on stimulation days 3, 4, and 5.

Carryover effects: the sub-acute carryover effect gradually improved over days in the shuffled CR DBS condition, achieving up to 21% improvement in the mUPDRS during stimulation days 3–5, while non-shuffled CR DBS fluctuated in the range of −12–4% change in the mUPDRS (Figure 2B). The day-to-day carryover benefits of shuffled CR DBS increased over days and reached 17.5% of improvement by stimulation day 5, carrying over for 1 day after 5 stimulation days. Non-shuffled CR DBS, however, produced a gradual worsening of motor signs over the 5 stimulation days that continued until the 3rd day after 5 stimulation days (Figure 2C). No carryover effect was observed with tDBS. The subacute and day-to-day carryover changes in mUPDRS associated with tDBS fluctuated in the range of −11.7–6.2%.

### Non-Human Primate B

**Figure 3:**
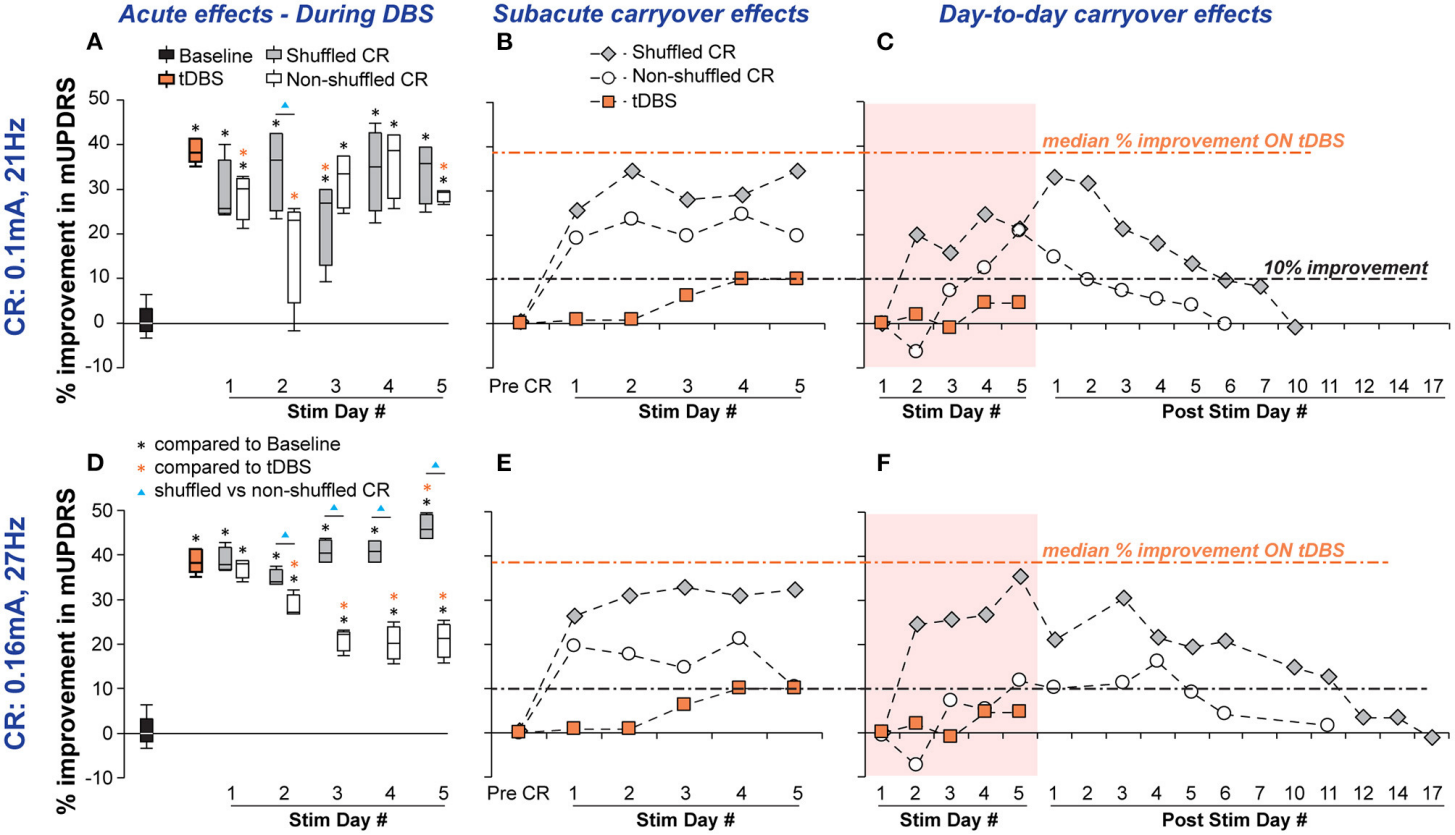
Acute and carryover improvements in mUPDRS acquired by shuffled and non-shuffled CR DBS in NHP B in two conditions: (i) CR DBS with a default stimulation intensity of 0.1 mA and burst frequency at 21 Hz (Top row) and (ii) CR DBS with the more effective stimulation intensity of 0.16 mA and burst frequency at 27 Hz (Bottom row). **(A,D)** Acute motor benefits across stimulation days measured by the percentage of improvement in mUPDRS compared with the baseline between shuffled and non-shuffled CR DBS were compared in each condition. Statistical comparisons were performed using the same method for NHP J (*, ▴*p* < 0.05). **(B,E)** Subacute carryover effects of CR DBS indicated by the percentage of improvement in the post-DBS mUPDRS. **(C,F)** Day-to-day carryover effects of CR DBS indicated by the percentage of improvement in the daily morning mUPDRS on stimulation and post-stimulation days.

Daily acute effects: with the default stimulation intensity and burst frequency, there was only a modest difference in acute improvement between the shuffled and non-shuffled CR conditions (Figure 3A). Shuffled CR produced acute effects at the same level of tDBS in 4 out of 5 stimulation days, but the acute improvement induced by non-shuffled CR was similar to tDBS for only 2 out of the 5 days (Figure 3A). With the more effective stimulation intensity (0.16 mA) and burst frequency (27 Hz), however, the impact of shuffling on acute improvement was more pronounced. Shuffled CR produced greater acute improvement than non-shuffled CR in 4 out of 5 days (Figure 3D). The difference between the two stimulation conditions gradually increased over stimulation days and on the last day of stimulation, shuffled CR DBS produced acute motor benefits over two times that of non-shuffled CR DBS. Less variation in the daily mUPDRS scores was observed with the more effective stimulation intensity and burst frequency with both shuffled and non-shuffled CR DBS. Notably, shuffled CR DBS with the more effective setting produced the same acute effect as tDBS on the first 4 stimulation days and greater improvement on day 5 while the acute effect of non-shuffled CR produced less improvement than tDBS on most stimulation days (Figure 3D).

Carryover effects: greater sub-acute carryover benefits were observed with shuffled CR DBS than non-shuffled CR DBS on all stimulation days. This finding was consistent between CR using the default and more effective settings of stimulation intensity and burst frequency (Figures 3B,E). Different from the accumulating effects we observed with shuffled CR DBS in NHP J, the sub-acute benefits with CR DBS in NHP B were already achieved on stimulation day 1. Benefits with shuffled CR DBS fluctuated around 30% improvement, while those with non-shuffled CR fluctuated around 20% improvement in days 1–4 decreasing to ~10% on day 5. The tDBS did not induce any sub-acute carryover benefits and the percentage of change in mUPDRS obtained 60 min after DBS on stimulation days fluctuated in the range of 0.7–10%.

With the default CR setting, shuffled CR for NHP B induced greater motor improvement and a longer day-to-day carryover effect than non-shuffled CR. Slightly over 33% of maximum day-to-day carryover motor improvement was achieved with shuffled CR compared with 21% of maximum improvement with non-shuffled CR (Figure 3C). Using 10% improvement as a threshold for carryover benefits, following the 5 days of daily stimulation shuffled CR induced 6 additional days of carryover benefit while non-shuffled CR only induced 2 days of carryover benefit. The difference between the effects of shuffled and non-shuffled CR DBS in NHP B became more pronounced once the more effective CR setting was used. With this setting, shuffled CR DBS produced day-to-day carryover motor improvement that peaked around 36% and persisted above 10% for 11 days after the 5 days of daily stimulation, while non-shuffled CR DBS produced day-to-day carryover improvement that peaked around 16% and only persisted for 4 days (Figure 3F). No day-to-day carryover effect was observed with tDBS indicated by the percentage of change in daily morning mUPDRS scores fluctuating in the range of −1.1–4.4%.

## Discussion

This study illustrates the dependence of CR DBS efficacy on defining the effective set of CR stimulation parameters for acute, sub-acute, and day-to-day carryover effects on motor signs. Our results provide preliminary evidence supporting our hypothesis that shuffled CR DBS produces greater therapeutic effects than non-shuffled CR DBS. In addition, the results provide further support for the beneficial effect of STN CR DBS. With the appropriate parameter setting, CR DBS produced acute therapeutic effects that were comparable with those produced by tDBS, while using only half the stimulation intensity (NHP B). Carryover benefits observed with shuffled CR DBS might allow further reduction in the stimulation time of CR DBS. Being able to reduce both the stimulation intensity and amount of time required for stimulation are advantageous in that they are likely to be associated with a lower probability of side effects and energy consumption, requiring fewer battery replacement and/or less frequent recharging for rechargeable systems improving the quality of life for patients with PD who have undergone DBS.

### Importance of Exploration in CR Parameter Space in the NHP Model of PD

Coordinated reset deep brain stimulation has a vast parameter space and some of these parameters have been found in modeling studies to have a significant impact on its effect, such as stimulation intensity (15), burst frequency (15), pausing between stimulation (11, 22), and shuffling (5). Systematic evaluation of these parameters is critical for the development and clinical implementation of CR DBS. As the evaluation of each CR setting is time consuming (days/weeks), evaluating all the critical parameters, while challenging in patients with PD, is achievable in the NHP model of PD (23). During this study, the significantly greater acute and carryover motor improvement observed in NHP J provided preliminary evidence of the important role of shuffling in CR. These results encouraged and supported the further development of the investigational IPG (Boston Scientific) to incorporate automatic shuffling in the CR pattern. Additional experiments in NHP B utilizing this new capability of automatic shuffling further demonstrated the importance of shuffling in CR DBS, providing greater acute motor improvement and longer carryover benefits. This study demonstrated the impact of shuffling stimulation patterns and the importance of identifying the role of individual variables to define the optimal setting for CR DBS.

### Potential Mechanistic Differences Underlying the Effect of Shuffled and Non-Shuffled CR DBS

Modeling studies have hypothesized that a non-shuffled CR stimulation pattern activates neuronal subpopulations in a phase-shifted manner resulting in a desynchronizing effect that underlies its therapeutic effect (14). Models incorporating spike-time-dependent plasticity (STDP), however, have indicated that prolonged non-shuffled CR stimulation may induce synchronization within each neuronal subpopulation compromising the desynchronizing effect (5, 17). Instead of the abnormal synchronization related to PD, this synchronization can be induced by repeating the same stimulation sequence thus stimulating each neuronal subpopulation at a fixed frequency. The phase-shifting effect on neuronal subpopulations might underlie the acute therapeutic effects of CR DBS similar to that of tDBS observed with non-shuffled CR during the first 1 or 2 days of stimulation (Figures 2A, 3D). With prolonged stimulation, however, the desynchronizing effect might be compromised as neuronal subpopulations become synchronized again as a result of using the same stimulation sequence. Such a phenomenon could also explain the stepwise worsening of motor signs we observed across stimulation days of non-shuffled CR in NHP J that carried over for 2 days. To provide additional evidence to either refute or support these theories, additional studies investigating the effect of different CR parameter combinations on behavior and corresponding changes in neuronal activity in the basal ganglia thalamocortical circuit will be necessary.

### Limitations and Future Directions

Although significant differences between the shuffled and non-shuffled CR DBS were observed in both NHPs, there are several limitations to this study. Additional exploration in the CR stimulation intensities and burst frequencies in NHP B, were not performed in NHP J. This was due in part to the amount of time required to study each CR DBS setting but mostly to the different device capabilities and their availability. Different shuffle times and duration of stimulation were used in each animal due to the different capabilities of the device that was available at the time of experiments and different daily stimulation durations required to achieve a stable therapeutic effect. Although we were able to compare the effect of shuffled and non-shuffled CR DBS on motor signs within each subject, the difference in shuffle time, daily stimulation duration, and other parameters could have contributed to the differences in CR effects on each animal. It will be important in future studies to perform systematic evaluations of CR DBS using different combinations of critical parameters across multiple subjects. Our sample size was limited and assessments were not blinded. In spite of these limitations, the results provide important data setting the stage for future studies to confirm and expand upon these findings using quantitative, objective measures. Although stimulation related side effects were not observed in this study, with either tDBS or CR DBS, future long-term preclinical and/or clinical studies will be needed to compare the incidence of side effects related to these two DBS approaches. The results of this study, if supported with future studies, will have a direct translational impact on future DBS programming approaches as the capability of delivering CR patterns can be incorporated into current and future DBS systems.

## Data Availability Statement

The raw data supporting the conclusions of this article will be made available by the authors, without undue reservation.

## Ethics Statement

The animal study was reviewed and approved by Institutional Animal Care and Use Committee.

## Author Contributions

JW, JV, and GM conceived and designed the experiments. HB contributed to the study design. SK and HB designed and developed the study-specific IPG firmware and software. JW, SF, LJ, SN, and JZ contributed to animal training and instrumentation. JW, SF, and SN acquired and analyzed the data. JW, LJ, and JV were involved in drafting the article. All authors have reviewed the article and approved the final version for submission.

## Funding

This study was supported by the National Institutes of Health (R01NS117822, P50NS098573, R01NS037019, and R37NS077657), a research grant from Boston Scientific, the Engdahl Family Foundation, and a fellowship award from the Parkinson's Foundation. These funding sources played no additional role in the collection, analysis, or interpretation of the data, and did not play a role in writing the manuscript or in the decision to submit the article for publication.

## Conflict of Interest

SK and HB are employees and stockholders of Boston Scientific Corporation. Boston Scientific provided salary support for SK and HB but played no additional role in the collection, analysis, or interpretation of the data, and did not play a role in writing the report or in the decision to submit the article for publication. JV serves as a consultant for Medtronic, Inc., Boston Scientific, Abbott, Surgical Information Sciences, Inc. He serves on the Executive Advisory Board for Abbott and the Scientific Advisory Board for Surgical Information Sciences and has an equity interest in Surgical Information Sciences. GM has served in the past as a consultant for Abbott (Formerly St. Jude Medical). The remaining authors declare that the research was conducted in the absence of any commercial or financial relationships that could be construed as a potential conflict of interest.

## Publisher's Note

All claims expressed in this article are solely those of the authors and do not necessarily represent those of their affiliated organizations, or those of the publisher, the editors and the reviewers. Any product that may be evaluated in this article, or claim that may be made by its manufacturer, is not guaranteed or endorsed by the publisher.

## Abbreviations

mUPDRS: modified Unified Parkinson's Disease Rating Scale
PD: Parkinson's disease
CR: Coordinated reset
DBS: deep brain stimulation
tDBS: traditional deep brain stimulation
STN: subthalamic nucleus
GPe/i: external/internal segment of globus pallidus
SN: substantia nigra
MPTP: 1-methyl-4-phenyl-1,2,3,6-tetrahydropyridine
IPG: implantable pulse generator.

## References

[1] Deuschl G Herzog J Kleiner-Fisman G Kubu C Lozano AM Lyons KE. Deep brain stimulation: postoperative issues. Mov Disord. (2006) 21(Suppl. 14):S219–37. 10.1002/mds.20957 16810719

[2] Krack P Batir A Van Blercom N Chabardes S Fraix V Ardouin C. Five-year follow-up of bilateral stimulation of the subthalamic nucleus in advanced Parkinson's disease. N Engl J Med. (2003) 349:1925–34. 10.1056/NEJMoa035275 14614167

[3] Saint-Cyr JA Trépanier LL Kumar R Lozano AM Lang AE. Neuropsychological consequences of chronic bilateral stimulation of the subthalamic nucleus in Parkinson's disease. Brain. (2000) 123:2091–108. 10.1093/brain/123.10.2091 11004126

[4] Tass PA. A model of desynchronizing deep brain stimulation with a demand-controlled coordinated reset of neural subpopulations. Biol Cybern. (2003) 89:81–8. 10.1007/s00422-003-0425-7 12905037

[5] Tass PA Majtanik M. Long-term anti-kindling effects of desynchronizing brain stimulation: a theoretical study. Biol Cybern. (2006) 94:58–66. 10.1007/s00422-005-0028-6 16284784

[6] Popovych OV Tass PA. Desynchronizing electrical and sensory coordinated reset neuromodulation. Front Hum Neurosci. (2012) 6:e00058. Available from: 10.3389/fnhum.2012.00058 22454622 PMC3308339

[7] Hauptmann C Tass PA. Therapeutic rewiring by means of desynchronizing brain stimulation. Biosystems. (2007) 89:173–81. 10.1016/j.biosystems.2006.04.015 17184901

[8] Tass PA Qin L Hauptmann C Dovero S Bezard E Boraud T. Coordinated reset has sustained aftereffects in Parkinsonian monkeys. Ann Neurol. (2012) 72:816–20. 10.1002/ana.23663 23280797

[9] Wang J Nebeck S Muralidharan A Johnson MD Vitek JL Baker KB. Coordinated reset deep brain stimulation of subthalamic nucleus produces long-lasting, dose-dependent motor improvements in the 1-methyl-4-phenyl-1,2,3,6-tetrahydropyridine non-human primate model of Parkinsonism. Brain Stimul. (2016) 9:609–17. 10.1016/j.brs.2016.03.014 27151601 PMC10226766

[10] Adamchic I Hauptmann C Barnikol UB Pawelczyk N Popovych O Barnikol TT. Coordinated reset neuromodulation for Parkinson's disease: proof-of-concept study. Mov Disord. (2014) 29:1679–84. 10.1002/mds.25923 24976001 PMC4282372

[11] Lysyansky B Popovych OV Tass PA. Desynchronizing anti-resonance effect of m: n ON-OFF coordinated reset stimulation. J Neural Eng. (2011) 8:036019. 10.1088/1741-2560/8/3/036019 21555848

[12] Tass PA Silchenko AN Hauptmann C Barnikol UB Speckmann E-J. Long-lasting desynchronization in rat hippocampal slice induced by coordinated reset stimulation. Phys Rev E Stat Nonlin Soft Matter Phys. (2009) 80:011902. 10.1103/PhysRevE.80.011902 19658724

[13] Lysyansky B Popovych OV Tass PA. Optimal number of stimulation contacts for coordinated reset neuromodulation. Front Neuroeng. (2013) 6:5. 10.3389/fneng.2013.00005 23885239 PMC3717521

[14] Lysyansky B Popovych OV Tass PA. Multi-frequency activation of neuronal networks by coordinated reset stimulation. Interface Focus. (2011) 1:75–85. 10.1098/rsfs.2010.0010 22419975 PMC3262242

[15] Manos T Zeitler M Tass PA. How stimulation frequency and intensity impact on the long-lasting effects of coordinated reset stimulation. PLoS Comput Biol. (2018) 14:e1006113. 10.1371/journal.pcbi.1006113 29746458 PMC5963814

[16] Zeitler M Tass PA. Anti-kindling induced by two-stage coordinated reset stimulation with weak onset intensity. Front Comput Neurosci. (2016) 10:44. 10.3389/fncom.2016.00044 27242500 PMC4868855

[17] Zeitler M Tass PA. Augmented brain function by coordinated reset stimulation with slowly varying sequences. Front Syst Neurosci. (2015) 9:49. 10.3389/fnsys.2015.00049 25873867 PMC4379899

[18] Elder CM Hashimoto T Zhang J Vitek JL. Chronic implantation of deep brain stimulation leads in animal models of neurological disorders. J Neurosci Methods. (2005) 142:11–6. 10.1016/j.jneumeth.2004.07.007 15652612

[19] Hutchison WD Allan RJ Opitz H Levy R Dostrovsky JO Lang AE. Neurophysiological identification of the subthalamic nucleus in surgery for Parkinson's disease. Ann Neurol. (1998) 44:622–8. 10.1002/ana.410440407 9778260

[20] Vitek JL Zhang J Hashimoto T Russo G Baker KB. External pallidal stimulation improves parkinsonian motor signs and modulates neuronal activity throughout the basal ganglia thalamic network. Exp Neurol. (2012) 233:581–6. 10.1016/j.expneurol.2011.09.031 22001773 PMC3536483

[21] Wang J Johnson LA Jensen AL Baker KB Molnar GF Johnson MD. Network-wide oscillations in the parkinsonian state: alterations in neuronal activities occur in the premotor cortex in parkinsonian nonhuman primates. J Neurophysiol. (2017) 117:2242–9. 10.1152/jn.00011.2017 28228579 PMC5461662

[22] Popovych OV Xenakis MN Tass PA. The Spacing Principle for Unlearning Abnormal Neuronal Synchrony. PLoS ONE. (2015) 10:e0117205. 10.1371/journal.pone.0117205 25714553 PMC4340932

[23] Vitek JL Johnson LA. Understanding Parkinson's disease and deep brain stimulation: Role of monkey models. Proc Natl Acad Sci USA. (2019) 116:26259–65. 10.1073/pnas.1902300116 31871164 PMC6936402

