# Supplementary material for: Shuffling Improves the Acute and Carryover Effect of Subthalamic Coordinated Reset Deep Brain Stimulation
Source: Front Neurol. 2022 Feb 18;13:716046. doi: 10.3389/fneur.2022.716046 (PMC8894645; doi:10.3389/fneur.2022.716046)
Supplement: Supplementary file 1 [file Table_1.DOCX]

**Supplementary Table 1. Statistical analysis for the acute effects in mUPDRS**

| **A. Wilcoxon test of baseline and 5 stimulation days** | | | | | | |  |
| --- | --- | --- | --- | --- | --- | --- | --- |
|  | | **χ^2^** | **DoF, N** | **p** |  |  |  |
| **NHP J** | ***tDBS*** | 17.3 | 1, 25 | <0.0001 |  |  |  |
|  | ***Shuffled CR*** | 42.8 | 5, 50 | <0.0001 |  |  |  |
|  | ***Non-shuffled CR*** | 26.7 | 5, 50 | <0.0001 |  |  |  |
| **NHP B** | ***tDBS*** | 16.9 | 1, 24 | <0.0001 |  |  |  |
|  | ***Shuffled CR*** | 25.1 | 5, 34 | 0.0001 |  |  |  |
|  | ***Non-shuffled CR*** | 25.4 | 5, 34 | 0.0001 |  |  |  |
|  | ***Shuffled CR*†** | 29.4 | 5, 34 | <0.0001 |  |  |  |
|  | ***Non-shuffled CR*†** | 29.3 | 5, 34 | <0.0001 |  |  |  |

† more effective stimulation intensity and burst frequency

| **B. Daily acute effect of CR DBS compared to baseline and tDBS (Steel’s Test with control)** | | | | | | | | | | | | | |  |
| --- | --- | --- | --- | --- | --- | --- | --- | --- | --- | --- | --- | --- | --- | --- |
|  |  |  | **Control = Baseline** | | | | |  | **Control = tDBS** | | | | | |
|  |  |  | **p value of stimulation day #** | | | | |  | **p value of stimulation day #** | | | | | |
|  |  |  | **1** | **2** | **3** | **4** | **5** |  | **1** | **2** | **3** | **4** | **5** | |
| **NHP J** | ***Shuffled CR*** |  | 0.3776 | 0.0023 | 0.0024 | 0.0113 | 0.0023 |  | 0.0007 | 0.2575 | 0.9320 | 0.9913 | 0.7831 | |
|  | ***Non-shuffled CR*** | | 0.0025 | 0.0075 | 0.0341 | 0.1777 | 0.9690 |  | 0.8039 | 0.1505 | 0.0401 | 0.0032 | 0.0007 | |
| **NHP B** | ***Shuffled CR*** |  | 0.0198 | 0.0197 | 0.0275 | 0.0198 | 0.0197 |  | 0.2455 | 1.0000 | 0.0282 | 0.8216 | 0.6216 | |
|  | ***Non-shuffled CR*** | | 0.0198 | 0.2973 | 0.0197 | 0.0197 | 0.0197 |  | 0.0282 | 0.0279 | 0.6216 | 1.0000 | 0.0279 | |
|  | ***Shuffled CR*†** |  | 0.0198 | 0.0198 | 0.0197 | 0.0196 | 0.0197 |  | 0.9977 | 0.1817 | 0.6216 | 0.6204 | 0.0279 | |
|  | ***Non-shuffled CR*†** | | 0.0088 | 0.0088 | 0.0088 | 0.0088 | 0.0088 |  | 0.9796 | 0.0276 | 0.0276 | 0.0276 | 0.0279 | |

| **C. Wilcoxon test for shuffled vs non-shuffled CR DBS** | | | | | | | | | | | | | | | | | | |
| --- | --- | --- | --- | --- | --- | --- | --- | --- | --- | --- | --- | --- | --- | --- | --- | --- | --- | --- |
| **Stim day #** | **NHP J** | | | | |  | **NHP B** | | | | |  | **NHP B †** | | | | |  |
|  | **1** | **2** | **3** | **4** | **5** |  | **1** | **2** | **3** | **4** | **5** |  | **1** | **2** | **3** | **4** | **5** |  |
| **χ2** | 11.4 | 11.5 | 11.5 | 4.7 | 11.5 |  | 0.1 | 4.2 | 1.3 | 0.1 | 1.4 |  | 0.1 | 5.4 | 5.5 | 5.5 | 5.4 |  |
| **DoF, N** | 1,16 | 1,16 | 1,16 | 1,16 | 1,16 |  | 1, 8 | 1, 8 | 1, 8 | 1, 8 | 1, 8 |  | 1, 8 | 1, 8 | 1, 8 | 1, 8 | 1, 8 |  |
| **p** | 0.0007 | 0.0007 | 0.0007 | 0.0302 | 0.0007 |  | 0.7728 | 0.04 | 0.2454 | 0.7715 | 0.2425 |  | 0.7715 | 0.0202 | 0.0194 | 0.0187 | 0.0202 |  |
|  | | | | | | | | | | | | | | | | | | |
